# Spontaneous Rupture of the Extensor Pollicis Longus in a Break-Dancer

**Published:** 2014-01-17

**Authors:** Grant D. Shifflett, Eugene T. H. Ek, Andrew J. Weiland

**Affiliations:** Hand and Upper Extremity Service, Hospital for Special Surgery, New York, NY

**Keywords:** break dancer, tendon rupture, extensor pollicis longus, wrist, trauma

Dear Sir,

We report an interesting case involving the spontaneous rupture of the extensor pollicis longus (EPL) tendon in a break-dancer. A 29-year-old male professional break-dancer presented to our institution with the chief complaint of right wrist pain and inability to extend his thumb. He reported insidious dorsal-sided wrist pain but had the acute onset of worsening pain and inability to extend his thumb following a performance the weekend prior. Physical examination confirmed rupture of the EPL with a positive lift-off test (Fig 1). The patient underwent surgical reconstruction of the EPL with standard transfer of the extensor indicis tendon. Intraoperative findings showed EPL tendon attenuation at the level of the radiocarpal joint, dorsal capsular hypertrophy, and a bony spur adjacent to Lister's tubercle at the base of the third dorsal compartment. At latest follow-up, the patient demonstrated a well-functioning tendon transfer with restoration of thumb retropulsion. He has returned to all previous activities, in particular break-dancing.

Traumatic and atraumatic rupture of the EPL tendon are well-described phenomena. Ruptures occur as a result of mechanical or biologic stress. Common mechanisms include fractures of the distal radius, rheumatoid arthritis, local injection of steroids, and repetitive motion activities at the wrist.^1^^-^3

Break-dancing is a unique art form, which places tremendous stress on the upper extremity, and musculoskeletal injuries are well documented in this patient population.^4^ The wrist is particularly stressed because so many moves involve utilizing the hand-wrist unit as a peg around which the body moves or balances (Fig 2). We hypothesize that this patient's rupture was related to a repetitive hyperextension moment across the wrist leading to dorsal capsular hypertrophy and bony spur formation leading to chronic attenuation of the tendon eventually resulting in an acute rupture. The patient's clinical history in association with the intraoperative findings suggest this, and surgical pathology was consistent with chronic tendinous attenuation. Surgeons should have a high index of suspicion in a break-dancer patient with dorsal-sided wrist pain and consider early activity modification to prevent further attenuation and possible rupture.

## Figures and Tables

**Figure 1:**
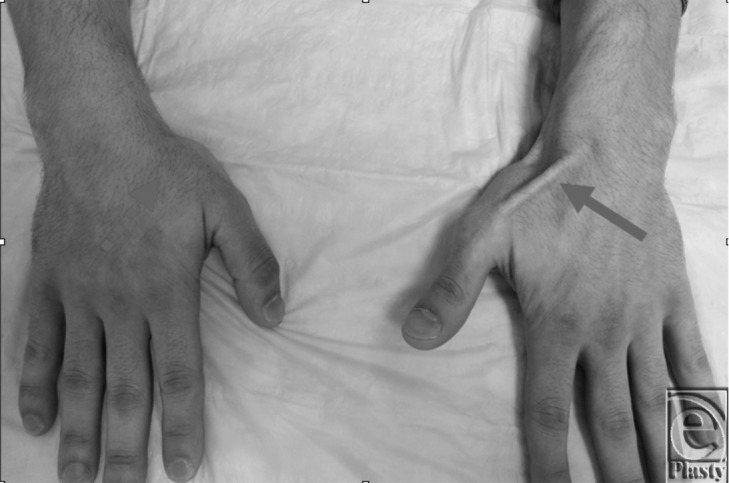
Clinical photograph showing attempted thumb retropulsion; solid arrow shows normal EPL tendon and dotted arrow shows absent EPL tendon. EPL indicates extensor pollicis longus.

**Figure 2:**
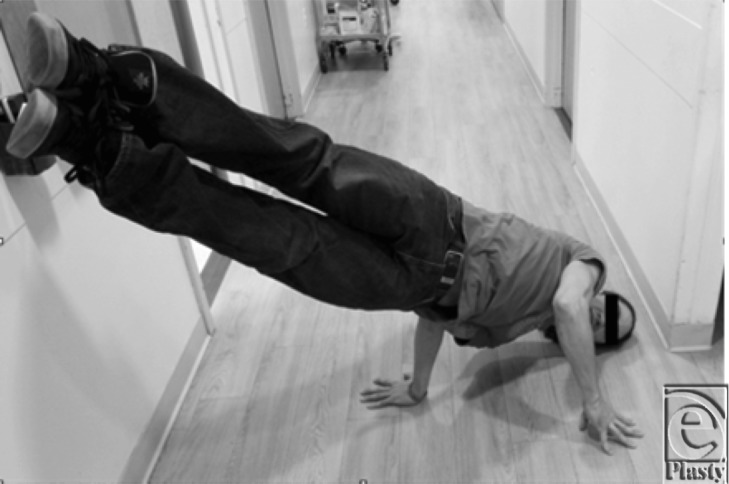
Patient demonstrating a common break-dancing “freeze” position, which places a tremendous amount of stress on the wrist.

## References

[1] Bjorkman A, Jorhsholm P (2004). Rupture of the extensor pollicis longus tendon: a study of aetiological factors. Scand J Plast Recon Surg and Hand Surg.

[2] Bonatz E, Kramer TD, Masear VR (1996). Rupture of the extensor pollicis longus tendon. Am J Orthop.

[3] Cho CH, Song KS, Min BW, Lee SM, Chang HW, Eum DS (2009). Musculoskeletal injuries in break-dancers. Injury.

[4] Rada EM, Shridharani SM, Lifchez SD (2013). Spontaneous atraumatic extensor pollicis longus rupture in the non-rheumatoid population. Eplasty.

